# Embodied Intelligence: Smooth Coping in the Learning Intelligent Decision Agent Cognitive Architecture

**DOI:** 10.3389/fpsyg.2022.846931

**Published:** 2022-04-06

**Authors:** Christian Kronsted, Sean Kugele, Zachariah A. Neemeh, Kevin J. Ryan, Stan Franklin

**Affiliations:** ^1^Department of Philosophy, University of Memphis, Memphis, TN, United States; ^2^Institute for Intelligent Systems, University of Memphis, Memphis, TN, United States; ^3^Department of Computer Science, University of Memphis, Memphis, TN, United States; ^4^Department of Philosophy, The University of Tennessee, Knoxville, Knoxville, TN, United States

**Keywords:** smooth coping, automatization, action selection, cognitive architecture, embodied cognition, global workspace theory, LIDA

## Abstract

Much of our everyday, embodied action comes in the form of smooth coping. Smooth coping is skillful action that has become habituated and ingrained, generally placing less stress on cognitive load than considered and deliberative thought and action. When performed with skill and expertise, walking, driving, skiing, musical performances, and short-order cooking are all examples of the phenomenon. Smooth coping is characterized by its rapidity and relative lack of reflection, both being hallmarks of automatization. Deliberative and reflective actions provide the contrast case. In Dreyfus’ classic view, smooth coping is “mindless” absorption into action, being in the flow, and any reflective thought will only interrupt this flow. Building on the pragmatist account of Dewey, others, such as Sutton, Montero, and Gallagher, insist on the intelligent flexibility built into smooth coping, suggesting that it is not equivalent to automatization. We seek to answer two complementary challenges in this article. First, how might we model smooth coping in autonomous agents (natural or artificial) at fine granularity? Second, we use this model of smooth coping to show how we might implement smooth coping in artificial intelligent agents. We develop a conceptual model of smooth coping in LIDA (Learning Intelligent Decision Agent). LIDA is an embodied cognitive architecture implementing the global workspace theory of consciousness, among other psychological theories. LIDA’s implementation of consciousness enables us to account for the phenomenology of smooth coping, something that few cognitive architectures would be able to do. Through the fine granular analysis of LIDA, we argue that smooth coping is a sequence of automatized actions intermittently interspersed with consciously mediated action selection, supplemented by dorsal stream processes. In other words, non-conscious, automatized actions (whether learned or innate) often require occasional bursts of conscious cognition to achieve the skillful and flexible adjustments of smooth coping. In addition, never-conscious dorsal stream information and associated sensorimotor processes provide further online adjustments during smooth coping. To achieve smooth coping in LIDA we introduce a new module to the LIDA cognitive architecture the Automatized Action Selection sub-module. Our complex model of smooth coping borrows notions of “embodied intelligence” from enactivism and augments these by allowing representations and more detailed mechanisms of conscious control. We explore several extended examples of smooth coping, starting from basic activities like walking and scaling up to more complex tasks like driving and short-order cooking.

## Introduction

In this article, we develop a conceptual model of smooth coping using LIDA (Learning Intelligent Decision Agent), a hybrid, embodied cognitive architecture implementing the Global Workspace Theory (GWT) of consciousness (Baars, 1988), the perception–action cycle (Neisser, 1976; Freeman, 2002; Fuster, 2004; Cutsuridis et al., 2011), grounded cognition (Harnad, 1990; Barsalou, 1999), appraisal theory (Lazarus, 1991; Roseman and Smith, 2001), long-term working memory (Ericsson and Kintsch, 1995), and other cognitive theories. It aims to be a “unified theory of cognition” (Newell, 1994), taking these and other disparate theories, and uniting them under a single, comprehensive architecture. LIDA is a conceptual and computational architecture that has been used as the basis for software and robotic agents. The current paper is the theoretical overview of how to implement smooth coping in LIDA. Following research will implement formalisms, code agents, and test the agents in various environments. We see this work as a first step toward robot implementation of smooth coping that will fit with current trends in robotics, such as learning by imitation (Bullard et al., 2019).

Smooth coping is the process of skillfully and adaptively acting, typically toward the completion of a task. Smooth coping covers a wide range of skillful behaviors, from those that are relatively basic like breathing or suckling, to those that are learned through painstaking training, as in becoming a pilot (Dreyfus and Dreyfus, 1980). Masterfully driving through traffic, skiing a slope, or running an obstacle course are all classic examples of smooth coping. However, the concept can also include cooking, herding sheep, dancing, tidying up, and many other activities in which it is possible to reach a state of optimized performance. The concept originates in phenomenological philosophy, particularly in the embodied phenomenologies of Heidegger, 1928/2010 and Merleau-Ponty, 1945/2012. Both of these thinkers were reacting against an intellectualized vision of human existence in philosophy and psychology that saw us as essentially epistemic agents geared toward knowing the world. As an alternative, they posited a vision of human existence that was, at its root, pragmatically oriented toward action and movement, and (for Merleau-Ponty) that was based in the agent’s embodiment.

In smooth coping the agent is not merely doing disjointed multitasking nor just doing automatized actions. Rather, most of the agent’s cognitive processes cohere toward fulfilling one distal intention. We outline how a LIDA agent might achieve smooth coping, and provide three case studies: walking, driving, and short-order cooking (see section “Conclusion”). Importantly, smooth coping in LIDA typically requires a “meshed” combination of conscious, consciously mediated, and never-conscious processes interwoven within a continuing series of cognitive cycles implemented using the Global Workspace Theory of consciousness (Franklin and Baars, 2010). Historically, in the LIDA conceptual model, Action Selection has only been able to choose one, and only one, action at a time. In this paper, we make a significant contribution to the LIDA model by introducing a new sub-module to Action Selection: Automatized Action Selection (AAS). This sub-module allows for concurrent selection of actions—AAS is capable choosing automatized actions in parallel. Furthermore, AAS runs in parallel with the original Action Selection algorithm which continues to choose one action at the time.

We begin by fleshing out recent debates on smooth coping and highlight the meshed nature of cognition supporting it (Christensen et al., 2016; Gallagher and Varga, 2020). We then introduce the LIDA model and the aspects of LIDA relevant to this project. For a more complete overview of LIDA, we recommend reading the tutorial and our two most recent papers (Franklin et al., 2016; Kronsted et al., 2021; Neemeh et al., 2021). We illustrate how smooth coping might take place in a LIDA agent by going through three case studies of increasing complexity: walking alone, driving in traffic, and short-order cooking (see section “Conclusion”).

## Smooth Coping

Although there has been a recent uptick in debates on smooth coping, the topic can be traced at least back to Aristotle and the notion of *phronesis* (typically translated as “practical wisdom”). Smooth coping debates since their earliest inceptions have typically been tied to culture and sociality—to smoothly maneuver the world is often to do so in rich social cultural contexts (Rietveld and Kiverstein, 2014). Thus, debates on smooth coping cut across discussions in social cognition, anthropology, performance studies, and discussions of “expert performance” (Cappuccio, 2019).

The crossover between motoric and cultural discussions when dealing with smooth coping is especially pronounced when looking at the phenomenological tradition. In the twentieth century, Martin Heidegger introduced the term *Zuhandenheit* in his monumental *Being and Time* (1927). Often translated as “readiness-to-hand,” *Zuhandenheit* refers to a mode of comportment that is pre-reflective and pre-theoretical. When I take something, let us say a tool like a hammer, as ready-to-hand, I am using it rather than reflecting on it. This usage is an embodied know-how rather than theoretical contemplation. Heidegger argued that the Western philosophical tradition focused exclusively on *Vorhandenheit* (“presence-at-hand”), that is, the theoretical comportment. For example, Kant’s theory of experience is explicitly aimed at supporting the endeavor of science. This focus on theoretical reason rather than embodied action is something we can see reduplicated in the history of artificial intelligence and robotics. In contrast, Merleau-Ponty, 1945/2012 examined embodiment and action as they dynamically interact with space, time, sexuality, other agents, and other domains. According to Merleau-Ponty, smooth coping is the most fundamental mode of our everyday lives. Years later, Hans Jonas (2001) developed a genetic phenomenology of subjectivity, according to which these basal strata of smooth coping enable higher-order cognitive processes to emerge, similar to contemporary claims of scaffolding. Across thinkers in the phenomenological tradition, we see an emphasis on embodiment in which smooth coping is a basic capacity of cognitive agents as they move through the world. In summary, many phenomenologists take the view that smooth coping forms the basic background of embodied human agency, and that more epistemically oriented, logical, or higher-order processes are less common and are founded against this background.

Building off of the phenomenological tradition, Dreyfus and Dreyfus (1980) developed a cognitive theory of smooth coping based on five stages of skill acquisition. According to their theory, expertise in a skill is characterized by automatization and a lack of higher-order thinking. On this model of smooth coping, experts have habituated their skills within a domain to the point that their movements are fully automatized. This, in turn, is supposed to explain why paying attention to oneself, or deploying higher-order cognitive processes, such as “strategizing,” can sometimes be detrimental to performance (Fitts and Posner, 1967; Cappuccio et al., 2019).

In the literature on smooth coping and expert performance, others have followed Dreyfus and Dreyfus and similarly argued that smooth coping in skillful action is a matter of complete automaticity (Papineau, 2013, 2015).

However, the Dreyfus model has in recent years been criticized by a variety of theorists, athletes, and artists, and from a variety of perspectives. For example, Barbara Gail Montero (2010, 2016) demonstrates that to be effective in many sports, the athlete must deploy both automatization and higher-order cognitive processes. Additionally, Montero et al. (2019) demonstrate that the empirical research program claiming that self-attention is detrimental to performance is based on flawed experimental design. Self-attention, monitoring, strategizing, and so forth, are often integrated into the flow of performance, rather than interrupting it.

The point here is that higher-order processes, such as planning, strategizing, monitoring, and so forth, are not always detrimental to expert performance, but on the contrary are often necessary for expert performance and successful smooth coping. Given this insight, smooth coping is often a matter of fluently integrating what some have called “online” (immediate sensory stimuli is needed) and “off-line” (detached from immediate sensory stimuli) cognition (Wilson, 2002). Several theories now propose an integrated web of causality between low-level and higher-order processes in expert performance and smooth coping more generally. Such models include “arch” (Høffding and Satne, 2019), meshed architecture (Christensen et al., 2016, 2019), the dual-process model (Neemeh, 2021), radically meshed architecture (Gallagher and Varga, 2020), and a variety of similar approaches (Bermúdez, 2017; Pacherie and Mylopoulos, 2021).

While these models vary with regards to their commitments, the general gist is the same: both low-level and higher-order cognitive processes are utilized and impact each other during expert performance. For example, automatized non-conscious processes, such as the continual adjustment of posture or dribbling of a basketball, can be impacted by higher-order conscious processes, such as thinking about and realizing the opponent’s strategy. A mixed martial arts fighter facing an opponent with a longer reach might strategically try to outsmart their opponent by trying to grapple rather than kicking and punching. Such a higher-order strategic decision in turn impacts how fighters adjust their postures and reconfigure their sensorimotor readiness toward certain action types.

In the literature on dance performance, some phenomenologists have similarly pointed out that even in highly choreographed performances in which one movement brings forth the next, expert dancers must adjust their performances to the particularities of the stage, that night’s audience, lighting, air density and humidity, costume malfunctions, and other factors (Bresnahan, 2014). In this same vein, and perhaps even more importantly, the expert dancer (and expert performer in general) must always move in and out of conscious monitoring of the body itself, to adjust in accordance with how the body feels that day (Ravn, 2020).

From these brief examples, we can see that embodied expertise, whether in mundane cases like walking or driving or in highly specialized domains, such as sports and performance, involves a fluent intermixing of various cognitive processes and different levels of awareness (conscious, never-conscious, pre-conscious, pre-reflective). While meshed architecture approaches differ on their commitments to concepts, such as “mental representation” or how to conceptualize the causation between different cognitive mechanisms, it is commonly agreed that smooth coping is not just a matter of automatization. Rather, we frequently utilize and change between various cognitive processes. For example, musicians sometimes report being in a state of complete automatization while simultaneously monitoring their own actions and the actions of fellow musicians. In such a state the musician playing is acting through automatization but they are ready to interject with top-down control at any moment (Høffding, 2019).

Similarly important in discussions of smooth coping and expert performance is the notion of dispositional skill or habit. Here thinkers tend to develop accounts of habits that are strongly inspired by John Dewey’s (1922) notion of habit as a context-sensitive, flexible, disposition to act. Whether working within explicitly anti-representationalist enactive cognitive science (Gallagher, 2020; Segundo-Ortin and Heras-Escribano, 2021) or representationalist cognitive science (Schack, 2004; Sutton et al., 2011; Bermúdez, 2017; Pacherie and Mylopoulos, 2021), there is a general agreement that habit is an important concept in expert performance and smooth coping. Habits in such a view are entrenched through practice but are flexibly adapted to a variety of contexts. Unlike motor programs that are contextually rigid (Ghez, 1985; Neilson and Neilson, 2005), habits are always regulated and finely adjusted by the current context—habits are ways of adaptively being in one’s environment (Dewey, 1922).

## The Learning Intelligent Decision Agent Cognitive Architecture

Learning intelligent decision agent is a systems-level cognitive architecture intended to provide a complete and integrated account of cognition (Franklin et al., 2016). Thus, rather than modeling one aspect of mind, the LIDA model aims to be a “unified theory of cognition” (Newell, 1994) capable of modeling human, animal, and artificial minds.^1^ Cognition, as it is used here, broadly encompasses every mechanism of mind including (but not limited to) perception, attention, motivation, planning, deliberation, metacognition, action selection, and motor control, as well as the embodiment of all of these activities. “Cognition” then is meant to cover the entirety of the agent’s mental life including its embodiment and embodied actions. Within the LIDA framework, “minds” are broadly conceived of as control structures for autonomous agents (Franklin, 1995; Franklin and Graesser, 1997). Here “control structures” (see Newell, 1973) are broadly conceived of as those mechanisms that allow an agent to pursue its agenda. To be an autonomous agent is in part to have an agenda, and to have a mind is to have structures that allow one to pursue that agenda (however simple or complex one’s agenda might be). Consequently, autonomous agents are always in the business of answering the question “What should I do next?”

Learning intelligent decision agent is composed of many short- and long-term memory modules, as well as special purpose processors called codelets. While modularity is sometimes seen as a “bad word” in contemporary philosophy of mind, the LIDA model is modular in the sense that it is composed of a collection of independent modules that are constantly performing their designated task. However, it is important to note that the LIDA model is *not* committed to the modularity of brains (Franklin et al., 2013). In fact, the LIDA model makes no claims about brains whatsoever. Thus, the LIDA model can be implemented even by brains that are dynamic and full of neural reuse (Kelso, 1995; Anderson, 2014).

Importantly, the LIDA model implements the Global Workspace Theory of consciousness (Baars, 1988, 2019). An agent typically cannot be aware of everything in its environment (external or internal) and therefore needs to “filter out” the most relevant information. LIDA agents therefore have information regarding the world “compete” for its attention in a module known as the Global Workspace. Whatever structure wins (most typically a coalition of structures) is globally broadcast to every module throughout the model—hence the term “the global broadcast.” In this way, the Global Workspace functions as a filter that dictates what information becomes available to the rest of the agent’s modules.

In LIDA, sensory stimuli are used to construct both a rich model of the external environment and an internal environment within the module known as the Current Situational Model (CSM). In broad strokes, the CSM creates a model of the world, and different parts of the model are then sent to compete in the Global Workspace.

The LIDA model utilizes two types of special purpose processors—structure building codelets and attention codelets. Structure building codelets build, potentially complex, representational structures in LIDA’s CSM. These structures can include, among other things, sensory content from an agent’s environment and cued long-term memories (e.g., from Perceptual Associative Memory, Spatial Memory, Transient Episodic Memory, and Declarative Memory). Attention codelets, on the other hand, continually monitor the CSM looking for structures that match their concerns. If found, pre-conscious content and its corresponding attention codelets are formed into *coalitions* that compete for consciousness in LIDA’s Global Workspace.

**Figure 1:**
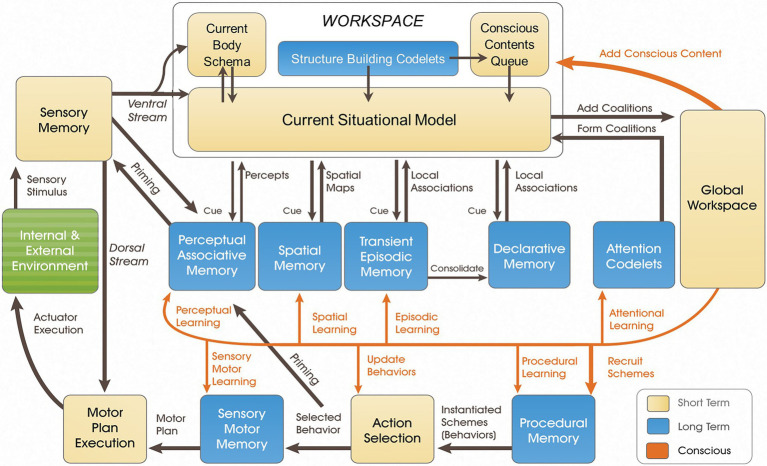
The LIDA model cognitive cycle overview diagram.

Coalitions consist of attention codelets and the contents for which they advocate. These coalitions are then sent to *compete* within the Global Workspace for conscious “attention.” The competition taking place within the Global Workspace module decides to what the system will consciously attend. Whichever coalition has the highest activation has its content broadcast to every LIDA module across the model (i.e., its content is *globally broadcast*). Consciousness consists of, among other things, the frequent serialized broadcast of discrete cognitive moments unfolding across overlapping cycles, that is then typically processed by each module. In other words. Consciousness is discrete and one thing after the other occurs at rapid pace (Baars, 1988). While all of LIDA’s modules take in input asynchronously, the serialized nature of the global broadcast facilitates a smooth serialized unfolding of consciousness and, as we shall see, of embodied action. For a general overview of the LIDA model, its modules, and processes, see Figure 1.

To be able to address the fact that agents have varying needs, across culture, personal history, and current situations, several variables are attached to structures in the CSM. For example, each structure has an activation value that is used in part to measure its salience. The salience of these structures is used to determine the activation of coalitions containing these structures, modulating their chance of winning the competition for global broadcasting in the Global Workspace. For an in-depth account of salience and motivation in LIDA (see McCall et al., 2020).

One of the core commitments of the LIDA research program is that the LIDA model is an embodied architecture (Franklin et al., 2013). This means that LIDA agents are biologically inspired in their design, and always in active commerce with their environments. In line with 4E approaches to cognition, LIDA agents are always in the process of answering the question “What do I do next?” Furthermore, constantly answering this question means that all LIDA agents have an “agenda” and in many embodied LIDA agents the agenda stems from the demands of the agent’s body.

Debates within embodied cognition often distinguish between weak and strong embodiment (Gallagher, 2011). In rough terms, an approach to cognition is weakly embodied if the body tends to simply be “represented” within a systems central processing. A system is strongly embodied if the arrangement of the systems physical body aids in the constitution of its cognition. However, the LIDA model does not neatly fit into this categorization. The LIDA model uses subsumption architecture (Brooks, 1991), and is in constant sensitive commerce with the environment through its dorsal stream. The LIDA dorsal stream, among other things, directly impact an agent’s physical involvement with its world. LIDA agent’s also have a body schema that constantly impacts the unfolding of sensorimotor action. At the same time, it is true that the LIDA model also represents its own body within the current situational model. Furthermore, the LIDA cognitive architecture is made so that it can be implemented both in physical and non-physical agents, such as robots or software agents, respectively. Therefore, the LIDA model contains both elements of strong and weak embodiment, and in physical agents, both approaches tend to be in play.

With this overview in hand, we are ready to dig into more detail regarding the LIDA cognitive cycle and action selection. Action selection is of special importance during smooth coping since successful smooth coping requires the skillful selection and execution of the right actions at the right time.

### The Cognitive Cycle

**Figure 2:**
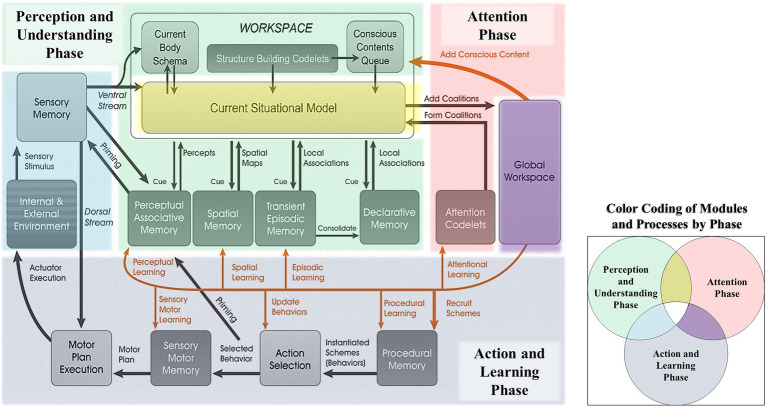
The LIDA Cognitive Cycle Diagram color coded. Green modules are involved in the perception and understanding phase, pink modules in the attention phase, and grey modules are involved in the Action and learning phase.

Learning intelligent decision agent’s cognitive cycle is divided into an understanding phase, an attention phase, and an action and learning phase (see Figure 2). LIDA’s cognitive cycle begins with external and internal sensory input, and the construction and updating of structures (i.e., representations) in the Current Situational Model (CSM). Structures that attract the attention of an attention codelet are then brought to the Global Workspace in which they compete for consciousness. The winning structure is broadcast throughout the model, and the system may make a decision to act (internally or externally) through an action selection mechanism. Learning can also occur as the result of each conscious broadcast. While a detailed discussion of learning in LIDA is beyond the scope of this article, it suffices to say that a LIDA agent typically learns with each cognitive cycle (as a direct result of its conscious broadcast).

For readers new to LIDA, it is helpful to remember that each cognitive cycle is rapid, lasting only 200–500 ms in humans (Madl et al., 2011), and that LIDA’s modules work largely asynchronously and independently of each other. As a result, cognitive cycles can “overlap.” For example, the “action and learning phase” from one cognitive cycle can occur concurrently with the “perception and understanding phase” of the next. Thus, while each cognitive cycle is conceptually divided into discrete, serial phases, it is rarely the case that an agent’s modules and processes are completely inactive.

### Action Selection

During the action and learning phase of each cognitive cycle, LIDA’s Action Selection module will typically select *behaviors* that specify executable (internal or external) actions. This process of action selection is needed for many reasons. For example, it may be the case that many behaviors can accomplish a task, although not all of them equally well. For example, a box might be moved by carrying it, pushing it with one’s hands, scooting it with one’s foot, or even pushing it with one’s head while crawling on all fours. In these cases, Action Selection facilitates the selection of the most situationally relevant and reliable of these behaviors. Furthermore, at any given moment, agents may have multiple, competing desires and goals. Action Selection facilitates the selection of behaviors that are more likely to lead to the most desirable outcomes. Finally, Action Selection coordinates the parallel selection of non-conflicting behaviors. Historically, Action Selection chose one, and only one, behavior at a time. In this paper, we enhance the Action Selection module to include an Automatized Action Selection sub-module (see Section “Smooth Coping in LIDA”) that allows for the selection of multiple, non-conflicting behaviors in each action selection event.

**Figure 3:**
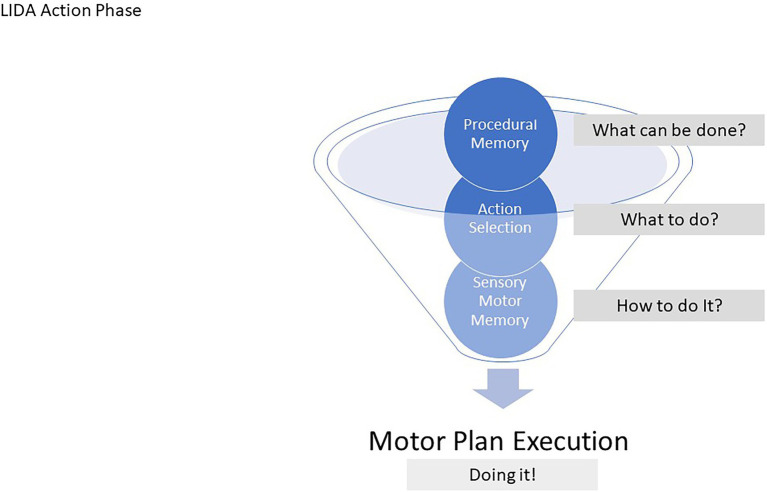
To gain a better grasp of the action selection process in LIDA, it is helpful to think of the process as a funneling toward specificity. Procedural memory contains information about things the agent can do under various circumstances at a somewhat abstract level. Action Selection, broadly speaking, chooses “what to do” in the agent’s particular circumstance. Sensory Motor Memory decides “how to do it” be picking a motor plan, high specificity, and Motor Plan Execution carries out the motor plan. In this way, actions are procedurally selected with increasing specificity.

Action Selection depends on LIDA’s Procedural Memory, a long-term memory module that determinates situationally relevant actions and their expected environmental consequences. In other words, Procedural Memory specifies what actions are available to take, and would happen if they were taken, while Action Selection determines what the agent will do given that knowledge (see Figure 3).

As conscious content is globally broadcast throughout all of LIDA’s modules, it is received by Procedural Memory, which uses the contents of the conscious broadcast to instantiate^2^ *schemes* that are relevant to that conscious content. Instantiated schemes are referred to as behaviors, which are candidates for selection by LIDA’s Action Selection module.

Each scheme consists of a *context* (i.e., environmental situation), an *action*, and a *result* (i.e., that action’s expected environmental consequences). These can be specified at many different levels of abstraction and generality. Each scheme also contains a *base-level activation*, which serves as an estimate of the likelihood that the scheme’s result will follow from its action when taken in a given context. For example, a generic “key turning scheme” might specify an action that corresponds to the bodily movements needed to turn a key, the context of being near a lock, and the expected result of that lock being unlocked. Each successful selection and execution of this scheme’s action (in the given context) will generally result in an increase in its base-level activation. Similarly, each failure will lead to a decrease in its base-level activation. If, as we might expect, this “key turning scheme” generally succeeds, then it will eventually have a high base-level activation. However, if its context were *underspecified*, for example if it did not limit “key turning” to when an agent is “near a lock,” then its action might be taken in inappropriate situations, leading to an unreliable scheme that often fails inexplicably. This unreliability would manifest in the scheme having a low base-level activation.

At this juncture it would be natural to ask, “Wait, is there a scheme for everything? Is there a coffee making scheme? A TV watching scheme? A CrossFit scheme?” First, we must understand that many schemes are culturally specific. A LIDA agent that is implemented in a car factory floor robot does not need a “cool handshake” scheme. However, an agent that exists in a culture in which different handshakes are integral to cultural fluency likely has schemes for different culturally relevant greetings.

Second, we must understand that complex actions are achievable through the execution of multiple simpler actions. For example, riding a bicycle consists of pedaling with both legs, steering, braking, scanning the environment, and much more. Historically in LIDA, the coordination of multiple actions into complex actions has been implemented as *streams of schemes* (see section “Behavior Streams and Skill”). As a result of these streams, LIDA agents do not need to learn unique schemes for every complex action. Rather, seemingly novel complex actions can be manifested through multiple preexisting schemes. In this way, LIDA achieves a form of “transfer learning” (Pan and Yang, 2009). To further facilitate the learning of complex actions, in this paper, we introduce the *hierarchical* organization of schemes (see section “Smooth Coping in LIDA”), which in conjunction with the automatized action selection of actions allows for fluid agential behavior.

When Action Selection chooses a behavior that specifies an *external* action (that is, one intended to modify an agent’s external environment), it passes it to LIDA’s Sensory Motor Memory for execution. If, on the other hand, the chosen behavior specifies an *internal* action (for example, one used to support mental simulation), it is sent to (or used to spawn) a structure building codelet that updates the Current Situational Model accordingly.

The selection of a behavior can also result in the creation of an *expectation codelet*. Expectation codelets are a type of attention codelet tasked with monitoring the Current Situational Model for content that matches the expected results of the agent’s recently selected behaviors. This temporarily biases an agent’s attention toward the environmental consequences of its recent actions, helping to produce a feedback loop between an agent’s actions and their results. Thus, in line with enactive and predictive approaches to cognition, action, perception, and prediction are intimately tied together in a feedback loop.

Research on smooth coping generally agrees that smooth coping consists of a series of automatic and consciously controlled actions, as well as both low-level sensorimotor activity and higher-order thought, such as strategizing or monitoring (Christensen et al., 2016; Montero, 2016; Høffding, 2019; Gallagher and Varga, 2020). In other words, smooth coping is a combination of ingrained and automatic processes with conscious and deliberate processes resulting in fluent and skillful action. In LIDA, this is modeled through the combination of four different modes of action selection: consciously mediated action selection, volitional decision making, alarms, and automatized action selection (Franklin et al., 2016, pp. 29–32).

Consciously mediated action selection refers to the many actions an agent performs in which the conscious broadcast is involved, while simultaneously being unaware of the selection processes that go into choosing those actions. For example, in sailing, the sports sailor might be consciously aware of the different ropes on the mast but is *not aware* of the competition in Action Selection that makes her choose the particular rope grip she ends up deploying. Similarly, a tennis player might be consciously aware of the ball as it approaches but is not aware of the action selection process that make him choose the smash over the volley.

Volitional action selection refers to the type of action selection in which the agent is consciously and actively aware of *some* of the selection processes. For example, when an agent is deliberating about what is the best move to make in a board game, and mulling over the different choices, outcomes, and pitfalls, they are doing volitional action selection. By mulling over different possible actions and their outcomes, “options” are created in the Current Situational Model (Franklin et al., 2016). Such options can become conscious and make their way to Procedural Memory, which may then instantiate behaviors based on these options. Action Selection may then choose from among these behaviors. Hence, the first part of volitional action selection is conscious while the second part is unconscious (the conscious broadcast is being utilized but the agent is not aware of the process taking place in Action Selection). In fact, in no mode of action selection is an agent aware of what is happening within the Action Selection module—the module just continuously does its job. In short, during volitional action selection, the agent is aware of the options they are juggling but *not aware* of what is going on “inside” Action Selection.

Alarms are never-conscious processes that bypass the competition in the Global Workspace. If some object or event is recognized by Perceptual Associative Memory as an alarm, the object or event will be sent straight to Procedural Memory to instantiate schemes. Behaviors relevant to alarm content are assigned a high activation value in Action Selection and are typically selected and immediately passed along to Sensory Motor Memory—which in turn passes along motor plans to Motor Plan Execution. Put simply, many agents have experienced acting in an alarming situation, and only becoming aware of their actions after the fact. For example, having a big spider climb on one’s arm for a lot of people will result in a series of brushing, jumping, and spasms, in which they are only aware of the threat after the fact. Similarly, in driving, many drivers experience reacting to dangerous situations as fast or faster than they are consciously aware of the situation. Note here that alarms can be both innate as in the spider example or culturally determined as in the driving example.

The final mode of Action Selection is automatized action selection. Automatized actions are overlearned actions where one action can be thought of as calling the next. Selection of automatized actions proceeds unconsciously, that is, selection does not necessarily need content from the conscious broadcast. These are typically the kinds of actions that have been practiced time and time again, and they can be performed without conscious thought. For example, walking on an empty sidewalk is a typical automatized action. It requires little attention, and the agent can simultaneously focus on other matters. In this paper, we go into detail regarding automatized action selection in Section “Smooth Coping in LIDA.”

While we go into details regarding automatization in section “Smooth Coping in LIDA” it is worth noting here a core difference between automatized action selection and alarms. Alarm actions revert back to normal functioning once the alarm action has been executed and does not call for further actions. In this way, alarms are a temporary interruption of whatever the agent is doing. Automatized actions on the other hand do not interrupt or take priority over normal processes in the system. Furthermore, automatized actions specify which actions are to proceed them from within the Automatized Action Selection module (more on this in section “Smooth Coping in LIDA”).

While in humans this whole process, starting with Procedural Memory, Action Selection, Sensory Motor Memory, and finally Motor Plan Execution, might seem long and laborious, it is important to remember that this process is extremely rapid. Each cognitive cycle typically happens within a few hundred milliseconds (Madl et al., 2011). Thus, when dealing with fast paced dynamic action, as is often the case in smooth coping, the overlapping cognitive cycles are more than sufficiently speedy to make adjustments and act on the fly. Furthermore, we must remember that Motor Plan Execution operates in parallel with all other systems, allowing for non-conscious adjustments to in-flight motor plans. Additionally, the LIDA Sensory Motor System is based on Brooks’s subsumption architecture (Brooks, 1991), allowing for rapid agent world interaction.

Similarly, to enactive and predictive processing approaches to mind, LIDA agents are always in the process of adaptively acting; We can say that LIDA agents are perpetually answering the question “What should I do next?” In LIDA, Action Selection continually chooses a behavior among candidate behaviors and sends them to Sensory Motor Memory (unless the action is to deliberate). This ensures that the agent is always in the process of acting to stay in an optimal adaptive relationship to its environment.

### Behavior Streams and Skill

Smooth coping involves “skill” and “optimal grip.” To have an optimal grip on an activity is to skillfully navigate that activity with fluency and ease (Merleau-Ponty, 1945/2012; Rietveld and Kiverstein, 2014; Bruineberg et al., 2021). Concepts, such as “skill” and “fluency,” often include being able to execute several actions in an uninterrupted fashion and adjusting those chains of movements to the dynamical real-time changes and demands of the situation (Nakamura and Csikszentmihalyi, 2014).

In LIDA, skill and fluency are, in part, implemented *via behavior streams*. Besides individual schemes, Procedural Memory also contains streams of schemes that can be instantiated. A stream of schemes is a stringed-together series of action schemes that can be collectively instantiated using contents from one or more global broadcasts. The entire instantiated stream of schemes is known as a behavior stream. Once a behavior stream has been sent to Action Selection the module can rapidly select one behavior at a time and pass each of these behaviors on to Sensory Motor Memory (which in turn passes on motor plans to Motor Plan Execution).

For biological agents smooth coping often involves a series of fluent actions. For example, dribbling a basketball, taking three long strides, and then jumping for the slam dunk can occur as one integrated, fluent series of movements. Furthermore, people rarely do just one thing at a time. The action selection process in LIDA, therefore, often involves Action Selection, rapidly picking behaviors from several behavior streams.

Historically, in the LIDA conceptual model, Action Selection has always picked *one*, and only one, action at the time. However, in biological agents, physical actions frequently overlap. Therefore, in this paper we are enhancing LIDA’s Action Selection to support the simultaneous selection of multiple actions. Specifically, in addition to the selection of actions one after another by our original action selection algorithm, we are also supporting the simultaneous selection of automatized actions. This is achieved by Action Selection’s new Automatized Action Selection sub-module. Developing this sub-module is one of the contributions of this paper.

For example, one can imagine the (haunting) scene of a circus clown riding a unicycle, juggling, and deliberately, maniacally laughing while performatively grinning its teeth. Such a performance requires multiple skilled actions overlapping at once. Even though Action Selection is constrained to choose only one behavior at a time, this does not mean that the *execution* of previously selected behaviors must be sequential. Furthermore, Action Selection can rapidly choose behaviors from multiple concurrent behavior streams, and pass them forward to Sensory Motor Memory for execution.

To be a skilled agent at some activity involves (among other things) having finely tuned, well-rehearsed behavior streams and motor plan templates that can be flexibly adjusted to the demands of the present situation. In LIDA, much of the “skilled” aspects of smooth coping is handled by Action Selection, Sensory Motor Memory, and especially Motor Plan Execution.

As a behavior is sent to Sensory Motor Memory, the system must create a motor plan—a highly concrete plan of bodily movement. Motor plans specify sequences of specific movement commands (the motor commands) that direct each of the agent’s specific actuators. Here an actuator simply means one of the physical parts through which an agent acts on the world. For example, a factory robot might only possess a single “arm” actuator. Human beings, on the other hand, have a great many more actuators.

Motor plans and their motor commands react and adapt to rapid incoming data from Sensory Memory through a dorsal stream (Neemeh et al., 2021) to guarantee that the agent’s actions are in synch with the most current state of the environment.

Often in smooth coping, an environment may change as an agent is acting on it. For example, being a sports sailor involves skillfully maneuvering the sails of a boat as the vessel is being bumped and rocked by erratic winds and currents. To skillfully complete motor plans during such dynamic situations motor plans constantly react to sensory information through LIDA’s dorsal stream as the agent is acting. An agent sailing might issue a motor plan to reach for a specific rope. However, as they are reaching the boat is rocked by a large wave. Instead of continuing the reach in the same fashion, updating the motor plan in real time through the dorsal stream ensures that the agent adjusts their reach, and still successfully grasps the rope.

### Affordances, Action-Oriented Representations, and Behavior Streams

Recent research on smooth coping cashes out much of the skillful interaction loop between agent and environment in terms of affordances and sometimes action-oriented representations (Milikan, 1995; Clark, 2016; Williams, 2018; Gallagher, 2020; Bruineberg et al., 2021; Kronsted, 2021a). Affordances and action-oriented representations are two very similar concepts. Affordances are typically defined as possibilities for actions that exist as a *relation* between an enculturated agent and the environment (Gibson, 1979/2013; Chemero, 2009). Significantly, affordances are ordinarily thought of as a non-representational concept. Action-oriented representations are very similar—but as implied in the name, they are a class of mental representations. Action-oriented representations are representations that also beckon or move the agent into action (Milikan, 1995; Ramsey, 2007; Clark, 2016; Kirchhoff and Kiverstein, 2019).

In LIDA we take a middle-ground approach by using representational affordances. LIDA affordances are conceptualized as representations within the system. For a recent account of how LIDA agents learn and use affordances (see Neemeh et al., 2021). Here it will suffice to say that as LIDA agents become enculturated and trained in various activities, they learn to perceive new affordances upon which they can react. As a LIDA agent gains increased skill, their perceptual system can detect increasingly more fine-grained affordances that can factor into the selection of increasingly fine-grained behavior streams.

There is a careful relationship between action, learning, behavior streams, and affordances. One of the aspects of LIDA that make the model stand out from other cognitive architectures is the “L”—Learning. LIDA agents technically speaking can “learn” something new with every cognitive cycle. With each global broadcast, almost all modules can be updated with content from the broadcast, and each module (including the various memory modules) can perform some function in light of that broadcast. For example, Perceptual Associative Memory might build new connections, Transient Episodic Memory might put together a new event, the Conscious Content Queue adds to the specious present, perhaps Procedural Memory starts building a new scheme, and much more. For a detailed account of learning in LIDA (see Kugele and Franklin, 2021).

**Figure 4:**
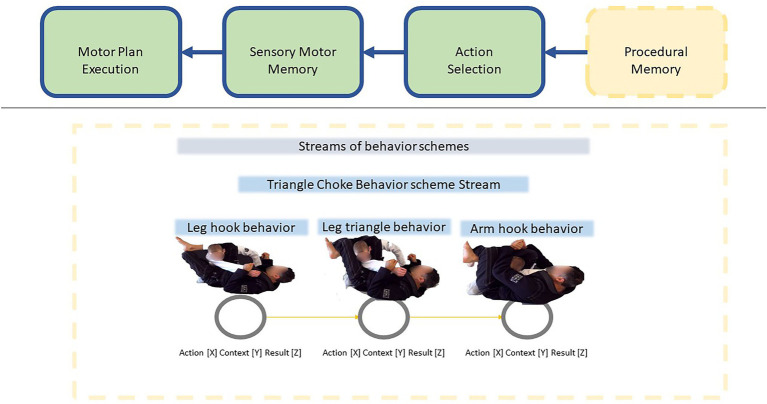
Procedural Memory contains streams of specialized behaviors. For example, to perform the Triangle Choke from Brazilian jiu jitsu the agent must first hook their leg around the opponent, form a leg triangle, and then tighten the triangle with legs and arm. These separate behaviors can be executed fluently by having each action linked together in a behavior stream that can have its variables specified with data from the conscious broadcast. By learning actions that are chained together, agents can execute highly specialized behaviors.

In terms of smooth coping, as a LIDA agent acts upon its environment, with each broadcast the agent slowly becomes more familiarized with that environment and the relevant task at hand. Such adaptation includes building more specialized and fine-grained affordances and behavior schemes for those affordances. For example, an agent might not know a thing about Brazilian Jujitsu, but with training, the different movements of opponents become associated with affordances for action or counter action (Kimmel and Rogler, 2018). An opponent going for the rear neck choke—affords putting one’s back flat on the mat. An opponent putting their weight in the wrong spot during close guard affords performing a leg triangle choke. There is a virtuous cycle between affordances and their associated behavior schemes. Smooth coping is most often a matter of having fine-grained affordances that make available the use of appropriately fine-grained behavior schemes (see Figure 4).

As agents perceives an event, they also perceive the associated affordances. If a coalition containing affordances wins the competition for broadcast in the Global Workspace, then the presence of the affordance in the broadcasted content will help instantiate behavior schemes, and thereby also promote winning the competition in Action Selection.

As mentioned earlier, choosing a behavior (perhaps from a behavior stream) also creates an expectation codelet to facilitate the monitoring of behavior-related outcomes. The creation of expectation codelets not only help bringing action outcomes to consciousness, but also helps ensure that the affordances associated with those action outcomes are also broadcast consciously. Acting on one affordance brings about the next affordance in an action promoting feedback loop. Such a feedback loop is in line with empirical and theoretical literature on affordances that conceptualizes smooth coping as a feedback loop between action and affordances (Di Paolo et al., 2018; Kimmel and Rogler, 2018; de Oliveira et al., 2021; Kimmel and Hristova, 2021; Kronsted, 2021b).

Overall, we see that smooth coping is not a matter of already being skilled at an activity. Rather smooth coping involves the ability to continually improve one’s skill and adaptivity. In LIDA, this adaptiveness is built into the flow of information across modules, facilitated by the conscious broadcast.

Of course, smooth coping is not only about knowing “what to do,” but also about having sufficiently developed sensorimotor coordination to do so—in layman’s terms having the right motor skills. Therefore, the skill cycle in LIDA also includes the agent building and refining increasingly sophisticated motor plan templates. Over many cognitive cycles, Sensory Motor Memory is slowly updated so that the agent is (hopefully) always in a position to know “how to do it” and with a great level of sophistication. Going into detail on how Sensory Motor Memory builds and updates motor plans is outside the scope of this paper. The important takeaway is that LIDA agents consistently update their action capabilities by updating their schemes for “what to do” (behaviors) *and* their plans for “how to do it” (motor plan templates).

Let us take the example of becoming better at sports—in this case, soccer. Through practice, soccer players learn to perceive the field and see it in terms of different opportunities. That is, the player, over time, learns to experience the game in terms of different affordances “in this situation, I can do a long pass, dribble past this guy on the right, or do a short backward pass.” Over time, players learn to see the field in terms of affordances that provide possibilities for “what to do” (potential behaviors). However, learning to exploit affordances is also a matter of learning how to concretely utilize the affordance “how to do it” (motor plans). With practice, agents therefore also fine-tune their physical capabilities in part by developing increasingly sophisticated motor plan templates—in the beginning, dribbling and kicking is clumsy, but over time it becomes second nature.

Naturally, doing something as advanced as expert level soccer requires multiple processes—some consciously mediated, others automatic. Hence, next, we will look at how different modes of action selection are interwoven during smooth coping, and the role of automatized action.

## Automatization and the Automatized Action Selection Sub-Module

One crucial aspect of smooth coping is that it involves both higher-level and lower-level cognitive processes (Christensen et al., 2016; Montero, 2016; Høffding and Satne, 2019; Gallagher and Varga, 2020). Let us return to the clown example. The clown performer who is simultaneously riding a unicycle, juggling, grinning, and talking to select audience members may utilize both consciously mediated, fully conscious, and automatized actions. Thus, to account for such overlapping in action during smooth coping, we need to take a look at how LIDA agents achieve automatization.

An automatized action is implemented as a series of behaviors in a behavior stream that have been mastered to the point in which those behaviors can be selected without mediation from the conscious broadcast—that is automatized behaviors can be selected without the need for sensory input updating. However, the execution of these behaviors may often require sensory input (for example over the dorsal stream or even the conscious broadcast).

For the purposes of smooth coping, it is often important that agents can do several actions simultaneously (for example, pedal and pass, dribble and tackle, punch and block, and the list goes on). In this paper, we therefore introduce a new sub-module to the LIDA model, namely, Action Selection’s Automatized Action Selection sub-module (AAS). This sub-module runs in parallel with Action Selection, and repeatedly sends behaviors to Sensory Motor Memory (SMM). For example, in our unicycling clown example, Automatized Action Selection can *repeatedly* choose the automatized behavior “pedal” and send it to SMM.

**Figure 5:**
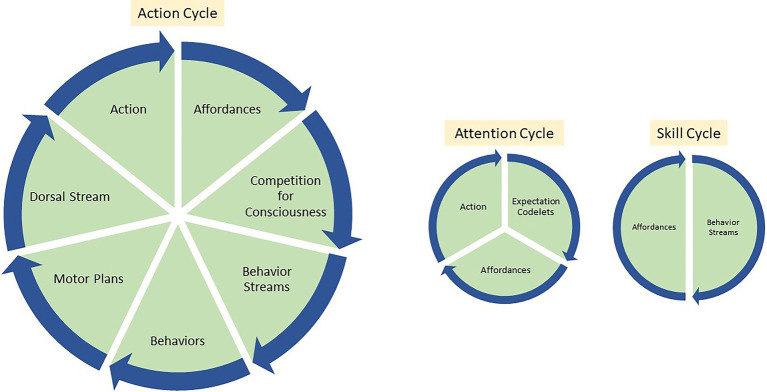
Above are three of the virtuous cycles in LIDA agent smooth coping. The first cycle demonstrates the affordance action cycle step by step. The second cycle demonstrates the relationship between expectation codelets new affordances and action. As an agent acts, they also generate expectation codelets and such codelets increases the chance of action-related affordances winning the competition for consciousness. Such biasing of attention in turn creates more actions. Finally, the skill cycle demonstrates how affordances lead to the creation of appropriate behavior schemes and executing behaviors in turn leads to the perception of new affordances.

**Figure 6:**
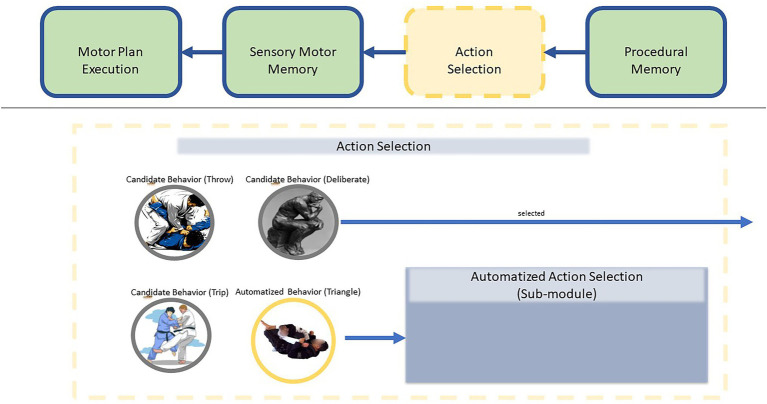
Here, we are zooming into Action Selection. In this case, Action Selection is choosing between a wealth of candidate behaviors. In this case, Action Selection chooses the “triangle choke” and passes it on to the Automatized Action Selection sub-module. Action Selection and the Automatized Action Selection sub-module run in parallel to facilitate multitasking. In this case, the agent is choosing to perform a Triangle choke while simultaneously choosing to “deliberate” on what to do next.

**Figure 7:**
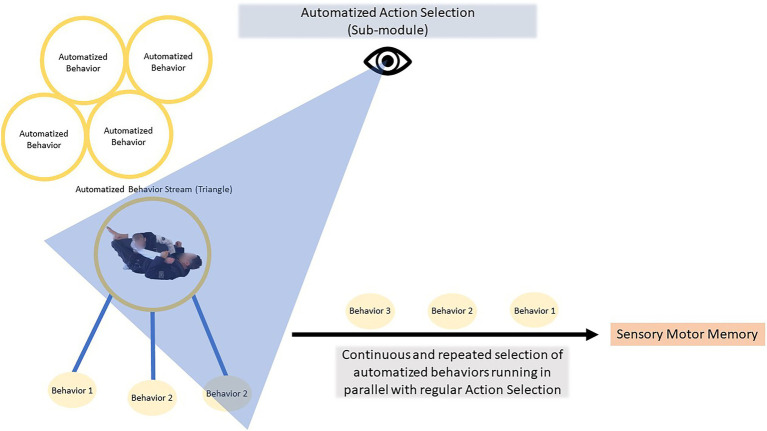
The Automatized Action Selection sub-module rapidly chooses one behavior at the time from candidate automatized behaviors (much like regular Action Selection). Like pearls on a string these behaviors are sent forward to Sensory Motor Memory at high speed; all in parallel with whatever might be happening in Action Selection. Differently from regular Action Selection selected automatized behaviors also “calls” for the next action to be selected to insure rapid smooth unfolding of the overlearned series of behaviors.

Having a sub-module that deals entirely with automatized behaviors, and being able to repeatedly select such behaviors, allows for Action Selection to focus in parallel on other forms of action selection, such as consciously mediated action selection or deliberation. Let us return to the example of Jiu Jitsu and the triangle choke. The “triangle choke” is a high-level behavior that consists of several movements (see Figure 5): leg hook, triangle hook, arm hook, and the squeeze. When Action Selection selects that high-level behavior, it sends that behavior to the AAS sub-module. From there AAS can select from the component behaviors in the “triangle choke’s” behavior stream. In short, Action Selection passes on high-level automatized behaviors to AAS, which then selects from lower-level component behaviors in the high-level behavior’s behavior stream. Being able to choose actions in parallel, allows for the Jiu Jitsu practitioner to carefully read their opponent’s patterns, and deliberate about what to do next while simultaneously producing complex behaviors, such as the “triangle choke” (Figures 6, 7). Smooth coping is often achieved by having Automatized Action Selection working harmoniously in parallel with other forms of action selection.

Automatized Action Selection runs in parallel with Action Selection choosing behaviors from automatized behavior streams (for example, walking, pedaling, dribbling, playing an ingrained song, etc.). Each of the behaviors from the selected behavior stream can be thought of as “calling the next” behavior in that stream. So once a high-level automatized behavior is selected, each of its lower-level behaviors, metaphorically speaking, gets to choose what behavior comes next. For example, if an agent is playing an overlearned piano piece (say *Alley Cat* by Bent Fabric) by way of Automatized Action Selection, each note, which corresponds to a lower-level behavior, “calls the next.” Once the first note has been chosen from the “*Alley Cat* Automatized behavior stream,” the first note selects the next note upon its completion. This produces the sensation recognized by many musicians as the piece essentially playing itself. This kind of automatization of one action calling the next also ensures that the musician can sing at the same time, lock eyes with the audience, playfully shimmy their shoulders, etc. all at the same time.

In LIDA technical terms, automatized behaviors are “degenerate” behavior streams—they are overlearned actions that *do not include branching options*. The lack of branching options is what allows the behavior to directly “call the next.” An automatized high-level behavior for pedaling may contain a behavior for pedaling with the right leg that then calls a behavior for pedal with the left leg—there are no branching options.

Importantly, automatized behavior streams can also be hierarchically structured where each of the behaviors in these streams can correspond to other behavior streams. This capability is critical because the specification of many actions benefits from hierarchical structure, and the reuse of these higher-level behaviors can be more efficient in memory. High-level behaviors often contain multiple behavior streams that must “line-up.” For example, to build a Reuben sandwich requires getting bread, mayo, sauerkraut, corned beef, and Swiss cheese, assembling the components, and putting them on a plate. Each of these sub-actions can be automatized and part of its own behavior stream. Collectively, these automatized behaviors contribute to realization of the high-level “Reuben sandwich” behavior.

A deli worker might make and wrap a sandwich like usual without taking the costumer’s difficult special order into account “only a little mayo, extra pickles, add sardines!” Making the sandwich differently requires consciously mediated action selection rather than automatization with one action calling the next. This explains why sometimes even when clearly intending to do one thing agents end up doing another because the beginning of the action was of an automatized nature.

It is important to note that although automatized behaviors do not have branching options and call the next action, they still generate expectation codelets. Just as with all other actions in LIDA, the generation of expectation codelets allow the system to keep track of the fulfilment of its actions so that the system may know whether to continue with its behaviors or switch to other behaviors.

As Automatic Action Selection feeds automatized behaviors forward to Sensory Motor Memory, that module can instantiate motor plans that also indicate the “timing” for how long the automatized action needs to be executed for—thereby mitigating the risk of doing something “mindlessly” for too long. In the music example, the motor plans for each note are designated a very short and precise timing. A motor plan for automatized “walking” on the other hand can have the temporal designation “until further notice” within the motor plan. We must remember that while automatization is often good for expert performance, smooth coping involves interwoven types of actions. Relying too much on automatization will often cause the task to fail.

## Smooth Coping in LIDA

**Figure 8:**
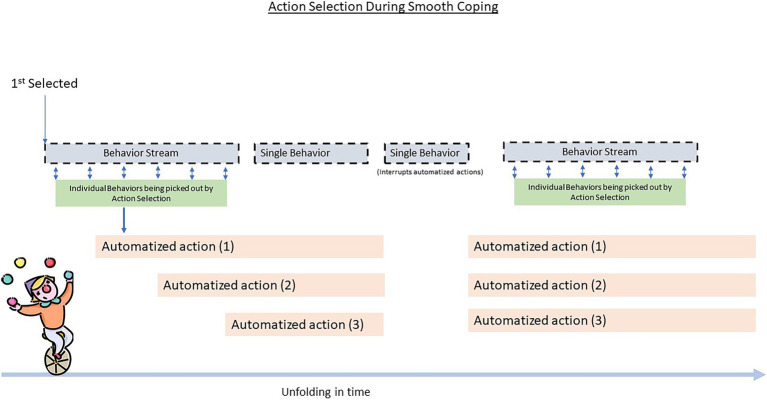
Here, we see an example of how an instance of smooth coping could unfold in a LIDA agent. The clown initiates automized actions, such as biking, juggling, and perhaps singing. In this case, the clown starts by biking, then overlays juggling, and finally starts singing (three concurrent automatized behaviors). Intermixed with these automized actions are behaviors picked out from a behavior stream and single behaviors. For example, the clown can turn its head toward select audience members and do a terrifying grin, perhaps do a spin on the bike or in the case of the single behavior that stops all other actions—do a backflip on the bike to then continue the routine.

One way to describe smooth coping is the use of automatization with intermittent use of consciously mediated actions (see Figure 8) as well as other overlapping action selection types toward the fulfillment of an intention (Kronsted et al., 2021). The agent is not simply multitasking or simply just doing automatization. Rather, all or most of the agent’s cognitive processes are cohering toward fulfilling one intention (completing this difficult recipe, football maneuvers, making it to work through traffic).

If some event forces the agent to abandon the cohering of their actions toward the intention the smooth coping process is interrupted. For example, the unicycling clown is engaging in smooth coping—cycling, juggling, grinning, and singing, all toward the intention of completing their act with a mesmerized audience. However, if a stagehand suddenly runs onto the stage and yells, “You must come at once, your wife is giving birth,” then the agent’s actions are no longer directed at the distal intention of finishing the act. Smooth coping has been interrupted. Less dramatically, if the phone rings while an agent is cooking, if the agent picks up the phone and attends to the phone call rather than the stove, smooth coping has been temporarily interrupted. The processes can, of course, be re-engaged as soon as the agent puts the phone down. In contrast, if the agent where to continue cooking while talking on the phone the agent can still be said to be smooth coping.

While we have here focused mostly on perception and action selection, and not memory processes, Smooth coping in LIDA is a phenomenon that operates across all modules. As mentioned previously in this paper we here introduce a new addition to the LIDA cognitive architecture—the Automatized Action Selection sub-module. In this section, we briefly go into more detail regarding the different modes of action selection, and then describe their interwoven nature during smooth coping especially in relation to the Automatized Action Selection sub-module. Finally, we provide three concrete case studies to demonstrate how the entire theoretical framework might play out (see section “Conclusion”).

### Interwoven Action Selection, and Feedback Loops

We can now see how action selection during smooth coping is achieved in LIDA agents through the interweaving of action selection types—consciously mediated action selection, volitional action selection, alarms, and automatized action selection.

As agents act in a variety of dynamically changing situations, they must deploy different forms of action selection to adaptively achieve their goals. For example, an agent might deploy a series of behaviors and behavior streams to carefully operate a table saw to carve pieces of wood in the right dimensions. Such behaviors and behavior streams might include walking to the table saw, grasping the wood, carefully lining it up on the table, and sliding the wood forward onto the saw while taking aim to ensure a straight-line cut. As the agent is deploying these behavior streams, they might also have intermittent moments of deliberation in which they actively think about which pieces to cut first and how to stack them up in the right order. The agent might further deliberate about the right dimensions of the cuts, which in turn will trickle down and affect the specifics of the instantiated motor plans and the execution of the actions in Motor Plan Execution.

Since the agent in our example is very skilled at carpentry, they have over years of practice developed automatized behavior streams and highly sophisticated motor plan templates for operating a table saw. So, the agent can operate the saw mostly through Automatized Action Selection.

Perhaps as the agent is working the table saw, their finger gets alarmingly close to the blade, and an alarm is triggered in the system pulling the hand backward. Alarms are importantly a part of the smooth coping flow when they enable the agent to continue with the intended activity. So, in the table saw example, the alarm that stops the agent from cutting off a finger naturally allows for the agent to continue the activity. However, an alarm to shake a large spider off one’s hand does not perpetuate the intended activity, and will typically break the smooth coping. The reason to bring up alarms here is to underscore that alarms usually must be learned, and are often skill and context-specific. For example, outside the context of Brazilian Jiu jitsu, getting a nice underhook hug is sweet and comforting. However, within the context of Jiu Jitsu it means the practitioner is about to be swept and likely lose the match. Hence, a context-specific alarm is likely triggered that will make the practitioner pull their arm back and try to close their armpits (to deny the opponent the underhook). Alarms are often an integrated part of mastering a skill since they are rapid and bypass the competition for conscious broadcasting.

Let us return to our table saw example. At some point over years of practice working the table saw has become automatized; the choosing of wood pieces, readying them at the table, and performing the cuts are now done by automatized behavior streams in which one action calls the next. In this way, the agent can repeatedly choose the same reliable behavior streams again and again until the job is done. Automatization allows for the selection of other actions (commonly, consciously mediated or deliberative actions) in parallel with the automatized action unfolding. The worker can operate the table saw (thanks to the Automatized Action Selection sub-module) while yelling at his/her apprentice to correct their form, bring them coffee, or perhaps deliberate about which technique to use for a difficult piece of wood that requires a different technique.

The overarching point is that smooth coping in LIDA involves deploying various forms of action selection each aimed at the task at hand. Be it alarms, consciously mediated actions, deliberative actions, or purely automated actions, each behavior selected coheres toward completing the agent’s goal in an adaptive fashion.

At this juncture, we cannot forget that smooth coping involves multiple feedback loops between the agent’s actions and changes in the environment. For example, driving behind a car while trying to read a funny bumper sticker on the car, involves having to be at the right range of distances to that car. Too far away and one cannot read the sticker, too close and the cars may collide—the agent must maintain “optimal grip” (Merleau-Ponty, 1945/2012; Dreyfus and Wrathall, 2014; Bruineberg et al., 2021). As already discussed, rapid dorsal stream updating of sensory information in movements updates Motor Plan Execution in action so that the agent can stay in an optimal relationship to their environment during action. There is a constant feedback loop between a LIDA agent’s actions and dorsal stream information.

Furthermore, with each action, an expectation codelet is also generated. As mentioned earlier, such codelets scan the Current Situational Model for objects and events related to the expected outcome of the agent’s actions. Structures brought to the Global Workspace by expectation codelets are typically highly salient and are very likely to win the competition for conscious broadcast. In this fashion, there is a feedback loop between an agent’s actions and their expectations. Through the feedback loop between actions and high activation results, LIDA agents can stay in careful attunement with the unfolding of their activities in dynamic contexts. We see that coinciding with an agent’s actions is attention toward the results of those actions which in turn help determine the completion of the intended activity. This is a biasing of attention toward the results of one’s actions which in turn helps perpetuate the completion of the intended activity.

Finally, the cognitive cycle in general assists in increasing adaptivity through learning. LIDA agents can update their memory modules with every cognitive cycle (Kugele and Franklin, 2021). In this way, the agent is always slowly but surely moving itself toward a greater degree of adaptivity.

**Figure 9:**
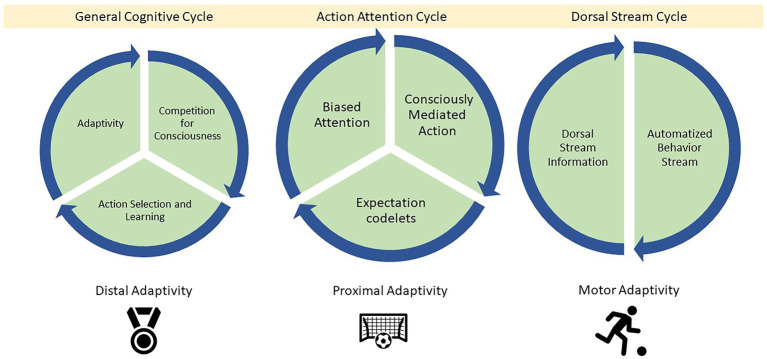
Here, we see three feedback loops that aid the agent across different timescales of smooth coping. The cognitive cycle in general aims to keep the agent in an equilibrium with its environment across long time scales. For example, winning a tournament. The attention cycle attunes the agent to their current context and the task(s) they are currently undertaking. For example, the context and task of playing and winning a soccer match. Finally, the dorsal stream cycle aims to keep the agent optimally adapted to their current task at the motoric level across rapid time scales. For example, dribbling, tackling, avoiding other players, shooting at the goal.

In general, we can think of at least three feedback loops that aid LIDA agents in smooth coping—the general cognitive cycle (adaptivity on a distal time scale), the action attention loop (adaptivity on a proximal time scale), and the action dorsal stream loop (motor adaptivity on a rapid timescale). In short, the cognitive cycle helps with task adaptivity over longer periods of time. Consciously mediated action selection aids in adaptivity in the agent’s current context. Automatization, motor plans, and the dorsal stream takes care of rapid in the moment adaptivity (see Figure 9).

We have looked at different forms of action selection and how they are interwoven toward the completion of a task during smooth coping. We have also looked at the different feedback loops that comes with these various forms of action selection, and how these feedback loops help the agent adapt to the task across different time scales.

## Discussion

For our discussion, we will apply everything we have looked at so far in three small case studies to see how smooth coping might play out in a LIDA agent in each scenario. We start with the relatively simple example of walking, and move up in complexity to driving, and then short-order cooking.

### Solo Walking

Sam wakes up at 5:00 am to take a daily walk in Shelby Farms Park. The path is a mile loop around a lake, and the early hour means that very few others are walking around at the same time.

Sam’s system utilizes the automatized behavior stream of walking. As the path curves ever so slightly around the lake, Sensory Memory updates Sam’s Motor Plans and motor commands so that Sam adjusts the direction of his body, the height and length of each step and other minor adjustments needed to move through the very accessible flat terrain. Minor differences in the height of the pavement mean that sometimes Sam’s Sensory Memory must update his stepping motor commands to be a little longer and a little higher.

Being mostly a matter of automatization, Sam can let his mind wander and think actively about other things in his life that need pondering (should I hop on the Bitcoin craze, is *Squid Game* really that good, what am I doing with my life?). Given that there are no obstacles in the terrain, Sam’s systems can simply continue to select and execute automatized walking behaviors. However, no automatized behavior is indefinite, and Sam does still need to periodically check for obstacles. Therefore, Sam still frequently looks at the road ahead and re-selects the automatized walking behavior.

Eventually, Sam notices a pedestrian and their dog approaching. The person and their dog have won the competition for consciousness, and Sam’s Action Selection is now choosing between multiple candidate behaviors (while Automatized Action Selection is making sure Sam is still walking). In Action Selection, walking onto the grass or standing still to let the dog and owner pass are the two most salient options. Standing still wins the competition in Action Selection, and Sam lets the person and their dog pass on the narrow path. Choosing this behavior also interrupts the automatized walking behavior.

An expectation codelet is generated looking, among other things, for a clear walking path since this is the expected outcome of Sam’s action. While the dog and owner are now behind Sam, the Current Situational Model continues to update. Then the expectation codelet brings the empty path structure to the Global Workspace to compete for broadcasting. Since Sam intends to walk and is expecting to have a clear path, the structure has high activation and may win the competition for consciousness.

As a result of the empty path coming to consciousness, Procedural Memory instantiates relevant schemes including a high-level “walking” behavior. This behavior and its behavior stream are sent to Action Selection. Action Selection chooses the highly relevant automatized “walking” behavior and sends it to the Automatized Action Selection sub-module. As a result, Sam keeps on walking with the Automatized Action Selection sub-module in charge of selecting actions. Now he is again free to continue to think about cryptocurrency, trending TV shows, and existentialism.

### Driving

Sam is done with his existential morning walk. At 8:00 am, Sam drives to work at a local diner. The route is a combination of suburban roads and highway driving, and takes approximately 20 min to complete. Some of the traffic is rush hour traffic.

Sam is utilizing an automatized behavior stream to follow the car in front of him at a safe distance. This of course also includes the motor plan for safe distance following which is receiving constant dorsal stream updating. Dorsal stream input to the motor plan makes sure that Sam does not push the gas pedal too hard or too softly. Following another car at the appropriate distance in rush hour traffic involves constant adjustment of motor commands to apply the right amount of pressure to the gas pedal.

However, since this is rush hour, Sam also needs to hit the brakes often and at the appropriate pressure. This means that through consciously mediated action selection, the behavior to press the brake is selected and executed at the appropriate level of pressure. Hence, Sam has an automatized car following behavior scheme and motor plan that is being frequently interrupted by the consciously mediated behavior of pushing the brake to remain at the right distance. Each time the brake has been pushed an expectation codelet is generated and helps the resulting distance between cars come to consciousness. The new distance between cars being broadcast in turn helps Action Selection either re-select the automatized follow behavior scheme, or perhaps some other automatized driving behavior.

*Via* consciously mediated action selection Sam decides to activate the behavior stream for changing lanes. Action Selection rapidly chooses each of the behaviors from the lane changing behavior stream. Sensory Motor Memory chooses between motor plans for each of the lane changing behaviors, and Motor Plan Execution begins carrying out the physical movements. In short Sam changes lanes; checks the back mirror, the side mirror, over the shoulder, turns on the blinker, checks again, turns the steering wheel left, turns the steering wheel back to neutral, rechecks windows and mirrors.

Suddenly a person who is texting and driving veers into Sam’s lane, and an alarm is triggered. The urgency of the situation means that the closing of the car bypasses the competition for conscious broadcast, and is sent directly to Procedural Memory. Schemes are instantiated and Action Selection chooses an appropriate behavior stream (break and veer). Given the urgency of the situation the break and veer behavior stream has very high salience, and easily wins the competition in Action Selection. Sensory Memory chooses appropriate motor plan templates and instantiates them, and Sam slams the breaks and veers the car away from the reckless driver.

Since an alarm was responsible for the avoidance maneuver, Sam has not yet realized what has just happened. Only approximately 100 milliseconds later, after the event has been recreated in the Current Situational Model, does Sam become “aware” of what just happened. However, during these 100 milliseconds, the break and veering maneuver takes place due to the rapidity of the alarm process. In this way, Sam survives the reckless driver.

During the alarm maneuver expectation, codelets were created, searching the Current Situational Model for the expected results of the dodging maneuver—a safe distance to the incoming driver. As this state of affairs obtains, Sam can now use consciously mediated action selection and choose to aggressively honk at the distracted driver—what a way to start your shift.

### The Short-Order Cook

Sam arrives at work a bit grouchy from the driving encounter. He begins his shift as a short-order cook at a diner. This diner has a counter with the short-order cook behind it and several tables. The diner is particularly busy for the first several hours of the day (people are coming in for brunch and hangover breakfast). Sam is engrossed in work throughout that time and is working on multiple orders simultaneously. The orders are coming in at a fast pace, and many guests are ordering modifications to their dishes (extra cheese, no cheese, chocolate chip pancake on the side, hot sauce on the side, side salad instead of fries, etc.) In addition to making the variety of menu items, several regulars arrive with their special orders and expect to be greeted as they sit down at the counter.

Let us begin with the first order—two eggs benedict, potatoes, and a side of halloumi salad (order one). Upon seeing the order slip, a distal intention is created in the Current Situational Model (finish order one)—this intention cues up information into the CSM regarding halloumi salad, potatoes, and eggs benedict. First, the intention (finish order one) wins the competition for consciousness, and in the next few cycles, structures regarding the current state of the kitchen and structures with information about eggs benedict, potatoes, and halloumi salad, each win a competition for consciousness (given the rapidity of cognitive cycles this is all still within the first second or two!).

At this point, information regarding the state of the kitchen and what to make are now present in the CSM and is broadcast to Procedural Memory. This information is now used to instantiate a multitude of schemes and scheme streams. These candidate behaviors are sent to Action Selection which must now choose “what to do.” In this case, the high-level action corresponding to the automatized behavior stream of poaching eggs is selected and sent to AAS. AAS selects behaviors from the “egg poaching” automatized behavior stream and sends them to the Sensory Motor Memory module. Sensory Motor Memory instantiates the chef’s highly skilled egg poaching motor plan, and sends it to Motor Plan Execution. This process continues with the other behaviors in the behavior stream being selected by the Automatized Action Selection sub-module where each action can be thought of as calling the next action. Thus, Sam ends up using automaticity to rapidly stir the vinegar–water mix, crack the eggs, and fish them back out.

As Sam is poaching eggs *via* automaticity, a regular customer sits down at the counter (Big Lu). The presence of the regular is highly salient to Sam, and easily wins the competition for consciousness. Procedural Memory upon receiving the global broadcast (containing the content of “Big Lu the regular”) instantiates several greeting behaviors, one of which is selected by Action Selection. Simultaneously, the egg poaching automatized behavior is still being executed. In other words, Sam is now stirring the pot rapidly with one hand, cracking eggs into the pot with the other hand, and directing his posture toward the customer while saying, “what’s up man, how you been?”

Big Lu tries to greet Sam over the counter with a handshake. But since Sam’s hands are full, he needs to use a compensating behavior. The outstretched hand comes to consciousness and instantiates several possible candidate behaviors—one such behavior is to use the elbow to complete the greeting. Choosing this behavior means that a motor plan is instantiated that also takes into account that Sam is still stirring a pot and cracking eggs *via* automaticity. As Sam reaches his elbow over the counter so that Big Lu can high-five his elbow, Sam’s motor plans for stirring and egg cracking can be radically adjusted through dorsal stream information and/or through subsequent conscious broadcasts.

As the eggs are being finished, a new order comes in: French toast and scrambled eggs with a side of bacon (order two). This fact comes to consciousness and creates a distal intention for order two which is stored for later retrieval in Sam’s Transient Episodic Memory as well as the CSM. Once Sam finishes order one, he can attend to and work on order two. However, at the moment, Sam still needs to assemble order one. The order two intention wins the competition for consciousness, and the intention is broadcast throughout the model, including various short and long-term memory modules (Sam is now working with two distal intentions present in the CSM).

However, Sam is still working on order one. So, Sam is now using consciously mediated actions to carefully assemble the eggs benedict for order one (he needs to grasp and assemble English muffin, ham, poached eggs, and hollandaise sauce).

Given that there are several chefs in the kitchen Sam does not have to make everything from scratch (for example, one worker is at the sauce station, another is at the meats stations). However, Sam does need to know where each component is and the location and activities of his co-workers. This information is updated in Sam’s Current Situational Model, including affordances in the environment. For example, if the lid is on the hollandaise pot, the sauce is not available for pouring. However, if the lid is at a tilt, Sam knows from engrained institutional knowledge that his co-worker is done with the sauce. In this case, the pot, therefore, affords “pourability” and Sam uses that information to perform a consciously mediated action of pouring some sauce onto the eggs.

As Sam is assembling the eggs benedict, pouring sauce, and adjusting the garnish, he is comparing the current state of the dish to long-term memory of what eggs benedict generally ought to look like—presentation is half the battle. Furthermore, as he is adding each component to the dish, expectation codelets are continually keeping his attention on track.

Sam puts the finished dish on the service counter for servers to pick up and begins order two, as orders three, four, and five arrive. As Sam is using automatized actions to make more eggs, flipping sauteed potatoes, or stirring, he is also keeping track of each order, and Action Selection is repeatedly sending new behaviors forward. Intermittent with the constant dance between automatized behaviors and consciously mediated behaviors, Sam might need to deliberate. For example, should Sam work on order five instead of four since not all the ingredients for four are ready? An ideomotor process begins with proposers, supporters, and objectors. “No, let us do the dishes in first come first order. That is easiest” “yes, let us put order four on hold to knock down the order we can while we wait for the salmon to finish cooking.” Even as Sam is actively deliberating, he is still executing both automatized actions and consciously mediated actions. Ultimately, skipping order four while the salmon is cooking wins the deliberation process, and Action Selection chooses behaviors relevant to making order five.

Around 4 pm the brunch rush is finally over, and Sam gets to hang up his apron and go home. What a day!

## Conclusion

Smooth coping is a common phenomenon in high skill activities, such as sports and performance, but also in our daily lives as we navigate the world. Smooth coping generally involves the cohering and centering of cognitive activity toward a task or activity (which is often highly culturally determined).

Learning intelligent decision agent agents engage in smooth coping by interweaving several forms of action selection including; consciously mediated action selection, volitional action selection, alarms, and automatization. Automatizations are overlearned behavior streams that allow for the selection of behaviors without conscious intervention; conceptually for one action to call the next. These automatizations also facilitate the concurrency of automatized action execution. Not only can automatized behavior streams be executed concurrently, but they can also be hierarchically structured. Smooth coping generally involves the biasing of attention and adaptivity toward tasks so that agents can gain an optimal grip on their various contexts. The LIDA model contains various feedback loops across distal, proximal, and rapid timescales that aid the agent in adaptivity. In line with recent embodied and enactive approaches to cognition, LIDA agents are constantly answering the question “what should I do next?” Through interwoven action and perception loops the agent pursues its agenda, and in the process reaches higher degrees of adaptivity across different time scales.

One strength of the smooth coping literature and our exploration of smooth coping in LIDA is that both expert action and quotidian life utilizes the same cognitive resources, and thus we can map a clear progression from novice to expert without the use of any additional “special” cognitive resources. In fact, from the literature on smooth coping and our overview of smooth coping in LIDA we can come to appreciate the complexity that goes into both expert performance and everyday cognition. Despite the ease at which it is performed, smooth coping is an immense achievement for any cognitive system be it artificial or organic.

## Author Contributions

All authors listed have made a substantial, direct, and intellectual contribution to the work and approved it for publication.

## Conflict of Interest

The authors declare that the research was conducted in the absence of any commercial or financial relationships that could be construed as a potential conflict of interest.

## Publisher’s Note

All claims expressed in this article are solely those of the authors and do not necessarily represent those of their affiliated organizations, or those of the publisher, the editors and the reviewers. Any product that may be evaluated in this article, or claim that may be made by its manufacturer, is not guaranteed or endorsed by the publisher.

## Footnotes

^1^For an overview of other cognitive architectures see Kotseruba and Tsotsos (2016).

^2^Instantiation is a specification process. It takes data structures and makes them more concrete. For example, in perception, the “template” for a chair could be instantiated into a specific chair, for example, a chair that is currently in front of an agent.
